# Inter-professional teamwork and its association with patient safety in German hospitals—A cross sectional study

**DOI:** 10.1371/journal.pone.0233766

**Published:** 2020-05-29

**Authors:** Julia Dinius, Rebecca Philipp, Nicole Ernstmann, Lina Heier, Anja S. Göritz, Stefanie Pfisterer-Heise, Judith Hammerschmidt, Corinna Bergelt, Antje Hammer, Mirjam Körner

**Affiliations:** 1 Medical Psychology and Medical Sociology, Medical Faculty, Albert-Ludwigs-University, Freiburg, Germany; 2 Department of Medical Psychology, University Medical Center Hamburg-Eppendorf, Hamburg, Germany; 3 Institute for Patient Safety, University Hospital Bonn, Bonn, Germany; 4 Center for Health Communication and Health Services Research (CHSR), Department for Psychosomatic Medicine and Psychotherapy, University Hospital Bonn, Bonn, Germany; 5 Occupational and Consumer Psychology, Institute of Psychology, Albert-Ludwigs-University, Freiburg, Germany; University of Adelaide, AUSTRALIA

## Abstract

**Background:**

Inter-professional teamwork is a prominent factor in quality of care and may lead to improved patient safety. Although team members’ points of view are highly relevant when trying to improve inpatient procedures, there is a lack of systematic assessment of their perceptions. Therefore, study aims were to explore inter-professional teamwork, safety-related behavior, and patient safety in German hospitals from team members’ point of view. Furthermore, we wanted to examine the association between inter-professional teamwork and safety-related behavior as well as the association between inter-professional teamwork and patient safety.

**Methods:**

We used cross-sectional pre-intervention data of a multicenter longitudinal study (German KOMPAS project). We gathered descriptive statistics for sample characteristics and to describe the current state of inter-professional teamwork, safety-related behavior, and patient safety. We used one-way variance analyses to assess differences between groups, and linear regression analyses to examine the association between inter-professional teamwork and the outcomes safety-related behavior, and patient safety.

**Results:**

326 inpatient care team members participated in the study. Participants perceived a moderate to high level of inter-professional teamwork, and a moderate level of patient safety. Moreover, they reached rather high values in safety-related behavior. Professional group, work experience, and period of employment had an impact on the perceptions of inter-professional teamwork, and patient safety. Higher inter-professional teamwork was associated with better patient safety. We did not find an association between inter-professional teamwork and safety-related behavior.

**Conclusions:**

Based on the association between inter-professional teamwork and patient safety, we recommend the implementation of team interventions. Because professional group, period of employment, and work experience had an impact on the perceptions of inter-professional teamwork and patient safety, we suggest future qualitative research to explore reasons for caregivers’ critical evaluation. Moreover, we recommend longitudinal studies to reveal causal relationships, and subsequently to determine areas of improvement for a safer health care.

## Introduction

Because medical progress is related to the growing specialization of medical professions and the fragmentation of workflows [1, 2], patient care is becoming increasingly complex [2–4]. Moreover, due to an ageing population, the spectrum of diseases leads to a rising number of multiple or chronic illnesses [5]. In addition, health care organizations are exposed to an increasing pressure of economic efficiency [6]. To accommodate these dynamics, inter-professional teamwork is indispensable and therefore a prominent factor of the quality of care [1, 7–11]. Inter-professional teamwork is defined as a *collaborative interaction* among at least two different health care professionals with various abilities and fields of activities to solve a shared task and reach a *common goal* [1]. Besides these criteria, inter-professional teamwork in inpatient care calls on *knowledge sharing* among the different health care professionals [4, 12]. Team members need to integrate their various perspectives to form a shared mental model [13], which allows the inter-professional team to adapt to changing task demands [14].

Studies showed that inter-professional teamwork may result in improved employee well-being [15], and employee satisfaction [16]. Furthermore, inter-professional teamwork fosters patient satisfaction [17, 18], and can lead to decreased length of stay [19], medical errors [20], and mortality [21] as well as to improved patient safety [5, 22–27], which is one of the national health goals in Germany [28], and one of the key issues of health care systems worldwide [25, 29]. Despite this relevance, studies found that inter-professional teamwork in inpatient care is still suboptimal and needs to be improved in order to ensure safe patient care [30–32]. To achieve this improvement the World Health Organization (WHO) stated the importance of inter-professional teams to acquire safety-relevant knowledge and skills [10]. Moreover they emphasized the relevance of safety-related behavior to improve patient safety [10]. According to Schwappach, Frank & Buschmann [33], safety-related behavior relates to attitudes, norms, perceived behavioral control, knowledge and potentially perception of risk and preventability.

In order to improve inpatient procedures, the point of view of members of inter-professional teams is highly relevant. To our knowledge, there has not yet been a systematic assessment of the current situation of inter-professional teamwork in German hospitals, and its association with safety-related behavior regarding inter-professional teamwork (hereinafter referred to as “safety-related behavior”), and subjectively perceived patient safety (hereinafter referred to as “patient safety). Therefore, the aim of this exploratory study was threefold: First, we sought to explore inter-professional teamwork, safety-related behavior, and patient safety in German hospitals from the point of view of inpatient care teams. Second, we aimed to investigate the association between inter-professional teamwork, and safety-related behavior in order to take the relevance of behavioral aspects as stated by the WHO into account. Third, we aimed to examine the association between inter-professional teamwork, and patient safety. The research questions are as follows:

1. How do inpatient care teams assess inter-professional teamwork, safety-related behavior, and patient safety in their wards?
2. Do participants’ assessment depend on their gender, age, profession, leadership role, work experience or period of employment?
3. What is the association between inter-professional teamwork and safety-related behavior?
4. What is the association between inter-professional teamwork and patient safety?

## Materials and methods

### Study design and recruitment

We conducted a multicenter longitudinal study, which was part of the German KOMPAS project (Piloting and evaluating feasibility of a training program to improve patient safety for inter-professional inpatient care teams) [34]. To assess primary and secondary outcomes, we collected data through an online survey provided by UNIPARK (QuestBack) or with a paper-based survey according to the local study coordinator’s preferences. We collected data at three points of assessment (pre-intervention, post-intervention three months after the training, follow-up six months after the training). For the present study we used cross-sectional pre-intervention data in only one key area of the KOMPAS project (teamwork) collected between July 2018 and February 2019. For more information on the study design of the KOMPAS project please see our study protocol [34].

The study included 39 inter-professional teams of different wards (ear, nose and throat wards; surgical wards; internal medicine wards; urology wards; gynecology wards; hematology wards; neurology wards; cardiology wards; orthopedic wards; psychosomatic wards) in 13 German hospitals that met the two criteria: inpatient care teams (1) with at least 10 members, and (2) with an inter-professional composition. As we used data of the KOMPAS project, we excluded emergency and intensive care due to high regimentations and standardized procedures in teamwork. Moreover, we excluded pediatrics, because patient involvement (another key area of the KOMPAS project) would not be comparable to other wards. A local study coordinator per ward (mostly ward manager) supported the research team regarding staff recruitment and data collection at their ward. The following inclusion criteria for study participants were applied: (1) member of an inter-professional inpatient care team (e.g., physician, nurse, therapist), (2) at least 18 years old, and (3) fluent in German.

### Measures

According to the definition of inter-professional teamwork, we used three validated measures to assess the aspects of *inter-professional teamwork*: to evaluate goal orientation, we used the Goal Orientation Scale from the abridged version of Questionnaire on Teamwork (FAT-K) [35]. The scale consists of four bipolar items (e.g., “The objectives of our team are clear.” versus “The objectives of our team are unclear.”), which are to be rated on a six-point Likert scale. High scores indicate a high degree of goal orientation. The scale has an internal consistency of α = .78 [35]. To assess collaboration between nurses and physicians, we applied the Collaboration Scale of the Revised Nursing Work Index (NWI-R) [36]. It consists of three items (e.g., “Nurses and physicians have good working relationships.”), which are to be rated on a four-point Likert scale ranging from 1 (disagree) to 4 (agree). High scores indicate a good collaboration between nurses and physicians. The scale has an internal consistency of α = .84 [37]. To assess knowledge integration problems, we utilized the German version of the Scale of Knowledge Integration Problems (WIP) [38]. The scale consists of eight items (e.g., “Team members are not prepared to consider other points of view.”), which are to be rated on a five-point Likert scale ranging from 0 (does not apply at all) to 4 (applies completely). Low scores represent low knowledge integration problems. The scale shows an internal consistency of α = .86 [38].

To assess *safety-related behavior*, we used a situational judgement test (SJT) [39–41]. We specified safety-related behavior as a person’s decision to act in a certain way (intended behavior). The SJT described a typical situation of dealing with a doubt regarding a diagnosis. Participants were asked to rank five given realistic behavioral options in the way they most likely would act, ranging from 1 (This behavior most closely corresponds to my reaction.) to 5 (This behavior corresponds least to my reaction.). We provide our SJT in S1 Fig. Based on the literature, the worst response in the outlined situation is to remain silent despite the doubt. The ideal response is to speak up, share the concerns with the team, and initiate clarification for further procedures. Speak up is typical for well-functioning teams with a high level of trust [42]. Based on results of an expert workshop with members of inter-professional teams (e.g., physicians, nurses, therapists), we scored the ideal sequence (= highest level of safety-related behavior) with 30 points (4*4+3*3+2*2+1*1+0*0), and the worst sequence (= lowest level of safety-related behavior) with 10 points (4*0+3*1+2*2+1*3+0*4). For easier interpretation, we transformed the score linearly, now ranging from 0 to 100.

To keep the survey feasible and comparable across participants, we assessed *patient safety* as subjectively perceived by members of inter-professional teams with a single item from the German Hospital Survey on Patient Safety Culture (HSPSC-D) [43] (“Please give your work area/ unit in this hospital an overall grade on patient safety”). Participants rated the item on a five-point Likert scale ranging from 1 (failing) to 5 (excellent).

### Statistical analyses

For our descriptive analyses, we calculated frequencies, means, and standard deviations (research question 1) as well as one-way variance analyses (ANOVA) to explore differences between groups (age, gender, profession, leadership role, work experience [time in years, in which participants worked in their profession], period of employment [time in years, in which participants worked in their hospital]) (research question 2). In addition, we calculated post hoc tests (Bonferroni) to further explore the differences between groups.

We ran linear regression analyses to examine the association between inter-professional teamwork and the outcomes safety-related behavior (research question 3), and patient safety (research question 4). In order to assess the strength of the relationships between these measures, and thus, to rule out multicollinearity, we first conducted Pearson correlation analyses (cut-off .7). Because patient safety was assessed with one item only, we also checked Spearman-Rho correlation coefficients for this outcome. Based on the findings for research question 2, we controlled for gender, age, and profession.

For the constructs of interest (FAT-K, NWI-R, WIP, patient safety, and safety-related behavior), we calculated intra-cluster correlation coefficients (ICC) to determine the proportion of the total variance explained by hospital ward affiliation. In the descriptive variance analyses, we adjusted for clustering by first calculating the variance inflation factor (VIF) from the ICC and then adjusting the F-values according to the VIF [44]. The VIF is the factor by which the total sample size needs to be increased, in order for a cluster design to reach the same statistical power as an individual design. We report adjusted F-values and degrees of freedom according to the number of clusters. In the inferential statistical analyses, we accounted for the variance between hospital wards by using cluster-mean centered data. For this, we subtracted the mean values of the hospital wards from the corresponding individuals’ mean values [45]. We added the results of the individual level analyses as sensitivity analyses. For the constructs of interest, missing data occurred in 65 of 1,640 responses (4%). We replaced missing data on a subscale level [46] using multiple imputation (seed 5000, 50 imputations, 100 iterations). We conducted inferential statistical analyses with a final data set of n = 324. Visual examination of the histograms and probability-probability plots suggested that residuals were approximately normally distributed. The significance level for all statistical tests was 5%. We analyzed data using IBM Statistics SPSS (Version 25 for Windows) [47].

### Ethics approval and consent

The project was approved by the ethics commissions at the three trial sites (Albert-Ludwigs-University of Freiburg: 4/16_170397, Friedrich-Wilhelm-University of Bonn: 329/17, Medical Association of Hamburg: MC-298/17).

Participation in the study was voluntary. There were no risks for participants. Non-participation bore no disadvantages. Furthermore, participants could withdraw their consent at any time without naming reasons. Consent for participation was obtained in written form.

## Results

### Sample

Local study coordinators in 13 German hospitals invited 860 members of 39 inter-professional inpatient care teams. The response rate was 38% (*N* = 330). We excluded four members of inpatient care teams from the analyses: two did not give consent to participate, and two did not provide data at the pre-measurement. The final sample consisted of 326 members of inpatient care teams. Table 1 summarizes the sociodemographics.

**Table 1:** Sociodemographic data of the sample (*N =* 326).

		n	%
Gender	Male	78	23.9
	Female	248	75.8
Age	< 30 years	98	29.7
	31–40 years	97	29.7
	41–50 years	69	20.9
	> 50 years	62	18.8
Profession	Physician	78	23.6
	Nurse	220	67.0
	others	28	8.5
Leadership role	Yes	69	20.9
	No	257	77.9
Work experience	< 3 months	10	3.0
	> 3 months < 1 year	15	4.5
	1 to 5 years	75	22.7
	> 5 years	226	68.5
Period of employment	< 3 months	10	3.0
	> 3 months < 1 year	35	10.6
	1 to 5 years	101	30.6
	> 5 years	180	54.5

### Inter-professional teamwork

We report the results of the variance analyses in detail in Table 2. A total of 317 participants completed the FAT-K [35] to assess goal orientation. The mean score was 4.79 (*SD =* 0.77). Participants reported the highest goal orientation for the item ‘identifying with the team’s goals’ (*M =* 5.03, *SD =* 0.98), and the lowest goal orientation for the item ‘clear formulation of the requirements for the work results’ (*M = 4*.57, *SD = 1*.*14*). According to the ANOVAs, nurses perceived a significantly lower goal orientation than physicians. However, when we adjusted for clustering, there was no difference between the groups. There were no differences between female, and male participants, age groups, leadership roles, work experiences, and periods of employment.

**Table 2:** Results of the variance analyses for inter-professional teamwork.

	Goal orientation (FAT-K)			Collaboration between nurses and physicians (NWI-R)			Knowledge integration problems(WIP)		
Intra-cluster correlation coefficient	0.21			0.32			0.22		
Group variable	Mean (Standard deviation)	Test statistic (dF), p-value	Adjusted test statistic (dF), p-value	Mean (Standard deviation)	Test statistic (dF), p-value	Adjusted test statistic (dF), p-value	Mean (Standard deviation)	Test statistic (dF), p-value	Adjusted test statistic (dF), p-value
Gender		*F*(1,316) = 0.07, 0.79	*F*(1,33) = 0.03,0.86		*F*(1,318) = 0.06,0.80	*F*(1,33) = 0.02, 0.89		*F*(1,317) = 0.19,0.66	*F*(1,33) = 0.07, 0.79
Male	4.78 (0.79)			3.06 (0.72)			1.59 (0.62)		
Female	4.80 (0.78)			3.08 (0.70)			1.55 (0.70)		
Age		*F*(3,314) = 0.63,0.60	*F*(3,31) = 0.23,0.88		*F*(3,316) = 1.58,0.20	*F*(3,31) = 0.43, 0.73		*F*(3,315) = 0.76,0.52	*F*(3,31) = 0.27, 0.85
< 30 years	4.72 (0.70)			2.96 (0.76)			1.63 (0.63)		
31–40 years	4.83 (0.79)			3.15 (0.64)			1.57 (0.68)		
41–50 years	4.78 (0.92)			3.16 (0.69)			1.47 (0.67)		
> 50 years	4.89 (0.71)			3.07 (0.70)			1.51 (0.76)		
Profession		*F*(2,315) = 5.62,**0.004**	*F*(2,32) = 2.03,0.15		*F*(2,317) = 5.95,**0.003**	*F*(2,32) = 1.64, 0.21		*F*(2,316) = 6.78,**0.001**	*F*(2,32) = 2.41, 0.11
Nurse	4.71 (0.81)			2.99 (0.75)			1.65 (0.70)		
Physician	4.91 (0.63)			3.24 (0.61)			1.34 (0.61)		
Other	5.18 (0.73)			3.35 (0.44)			1.42 (0.58)		
Leadership role		*F*(1,315) = 3.31,0.07	*F*(1,33) = 1.19, 0.21		*F*(1,317) = 1.48,0.23	*F*(1,33) = 0.41, 0.53		*F*(1,316) = 0.52,0.47	*F*(1,33) = 0.19, 0.67
Yes	4.94 (0.66)			3.17 (0.73)			1.50 (0.70)		
No	4.75 (0.80)			3.05 (0.70)			1.57 (0.67)		
Work experience		*F*(3,313) = 0.49,0.69	*F*(3,31) = 0.18, 0.91		*F*(3,315) = 4.61,**0.004**	*F*(3,31) = 1.27, 0.30		*F*(3,314) = 1.80,0.15	*F*(3,31) = 0.64, 0.60
< 3 months	5.06 (0.30)			3.74 (0.36)			1.11 (0.36)		
> 3 months < 1 year	4.91 (0.40)			3.07 (0.46)			1.38 (0.52)		
1 to 5 years	4.76 (0.69)			2.90 (0.75)			1.58 (0.59)		
> 5 years	4.79 (0.83)			3.11 (0.70)			1.59 (0.72)		
Period of employment		*F*(3,313) = 1.02,0.39	*F*(3,31) = 0.37, 0.78		*F*(3,315) = 3.66,**0.01**	*F*(3,31) = 1.01, 0.40		*F*(3,314) = 4.70,**0.003**	*F*(3,31) = 1.67, 0.19
< 3 months	5.15 (0.36)			0.91 (0.43)			0.91 (0.43)		
> 3 months < 1 year	4.88 (0.63)			1.36 (0.64)			1.36 (0.64)		
1 to 5 years	4.74 (0.74)			1.57 (0.61)			1.57 (0.61)		
> 5 years	4.79 (0.84)			1.62 (0.71)			1.62 (0.71)		

Statistically significant results are highlighted in bold.

A total of 316 members of inpatient care teams completed the NWI-R [36] to assess the collaboration between nurses and physicians. The mean score was 3.08 (*SD =* 0.70). Participants reported the highest collaboration for the item ‘good working relationships’ (*M =* 3.20, *SD =* 0.72), and the lowest collaboration for the item ‘exchange between nurses and physicians’ (*M =* 2.98, *SD =* 0.79). According to the ANOVAs, nurses scored significantly lower than physicians. Moreover, we found that groups significantly differed in their perceived collaboration depending on their work experiences, and periods of employment. We again adjusted for clustering and found no differences between the groups. The post hoc test suggested that there was a difference in perceived collaboration between participants who worked in their profession for less than three months and those who worked in their profession between 1 to 5 years (*p =* .004). The post hoc test suggested differences between participants who were employed in the hospital for less than three months, and for those who were employed between 1 and 5 years (*p =* .02), and for more than 5 years (*p =* .03). There were no differences in collaboration between female, and male participants, age groups, and leadership roles.

A total of 313 participants completed the WIP Scale [38] to assess knowledge integration problems. The mean score was 1.56 (*SD =* 0.68). Participants reported the highest knowledge integration problems for the item ‘different concepts to accomplish a task’ (*M =* 3.10, *SD =* 0.93), and the lowest knowledge integration problems for the item ‘unclear methods of other professions’ (*M =* 2.28, *SD =* 0.854). According to the ANOVAs, nurses perceived significant higher knowledge integration problems than physicians. In addition, we found that groups significantly differed in their perceived knowledge integration problems depending on their periods of employment. Again, adjustments for clustering resulted in no differences between the groups. The post hoc test suggested differences between participants who were employed in the hospital for less than three months and for those who were employed between 1 and 5 years (*p =* .02), and for more than 5 years (*p =* .01). There were no differences between female, and male participants, age groups, leadership roles, and work experiences.

### Safety-related behavior

**Fig 1:**
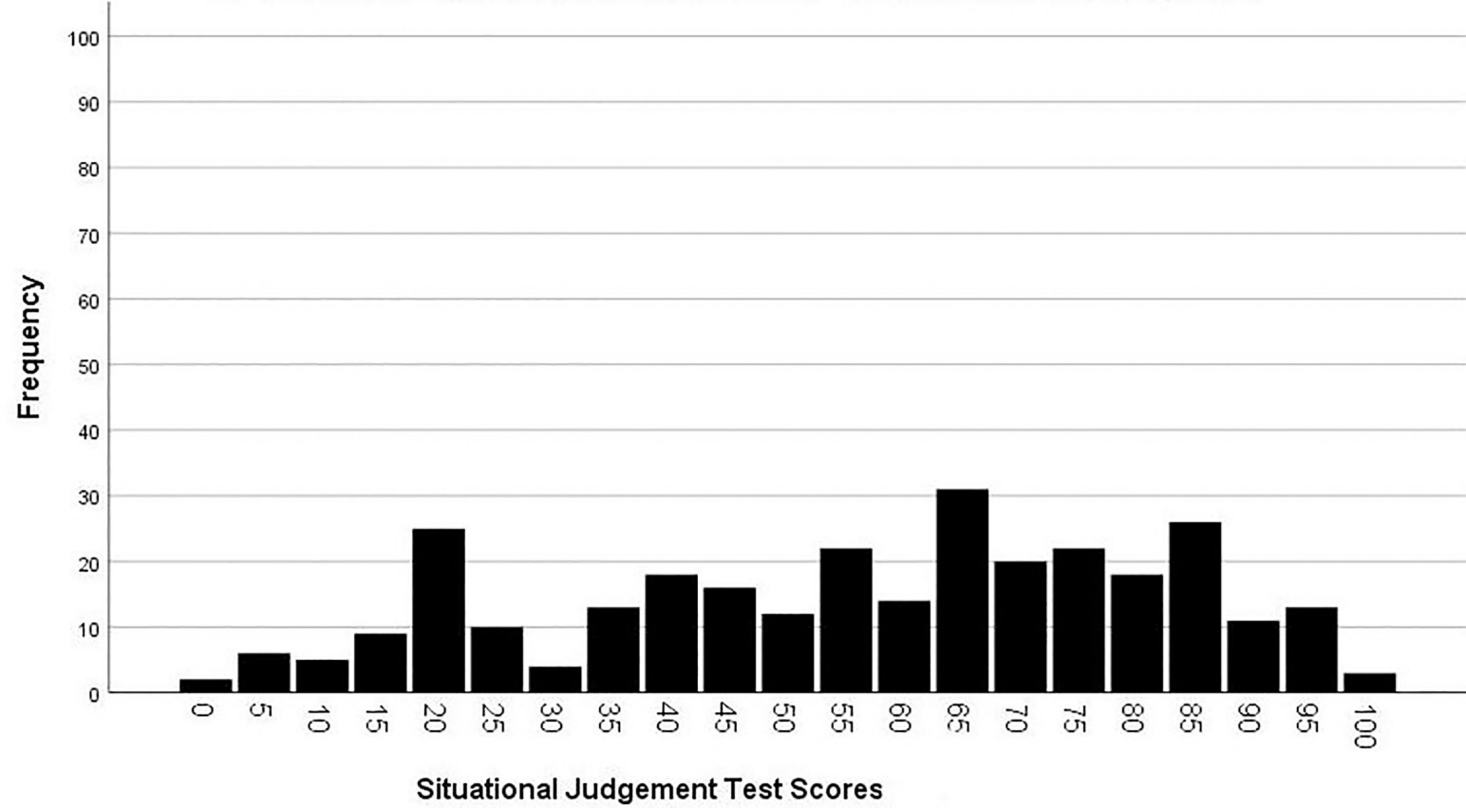
Scores in the SJT on safety-related behavior.

The SJT was completed by 91.5% of the sample (N = 300). The mean score was 56.23 (*SD =* 25.21), the median was 60, and the 25- and 75-percentile were 40 and 75 (Fig 1).

According to the ANOVAs, men scored significantly higher than women. There were no differences with regard to age groups, professions, leadership roles, work experiences, and periods of employment (Table 3).

**Table 3:** Results of the variance analyses for safety-related behavior, and patient safety.

	Safety-related behavior (SJT)			Patient safety (HSPSC-D item)		
Intra-cluster correlation coefficient	0.00			0.13		
Group variable	Mean (Standard deviation)	Test statistic (dF), p-value	Adjusted test statistic (dF), p-value	Mean (Standard deviation)	Test statistic (dF), p-value	Adjusted test statistic (dF), p-value
Gender		*F*(1,298) = 4.41,**0.04**	*F*(1,33) = 4.41, **0.04**		*F*(1,317) = 0.10, 0.75	*F*(1,33) = 0.05, 0.83
Male	61.69 (23.54)			3.42 (0.80)		
Female	54.54 (25.53)			3.39 (0.69)		
Age		*F*(3,296) = 1.13,0.34	*F*(3,31) = 1.13,0.35		*F*(3,314) = 2.23,0.09	*F*(3,31) = 1.08, 0.37
< 30 years	56.26 (25.97)			3.45 (0.66)		
31–40 years	59.23 (25.53)			3.50 (0.74)		
41–50 years	56.23 (22.83)			3.27 (0.77)		
> 50 years	51.40 (25.84)			3.27 (0.66)		
Profession		*F*(2,297) = 0.48,0.62	*F*(2,32) = 0.48,0.62		*F*(2,315) = 14.06,**<0.001**	*F*(2,32) = 6.82,**0.003**
Nurse	55.39 (24.71)			3.26 (0.71)		
Physician	58.80 (26.71)			3.74 (0.57)		
Other	55.77 (25.52)			3.50 (0.75)		
Leadership role		*F*(1,298) = 3.14,0.08	*F*(1,33) = 3.14,0.09		*F*(1,315) = 3.50,0.06	*F*(1,33) = 1.70,0.20
Yes	26.09 (3.26)			3.54 (0.68)		
No	24.86 (1.62)			3.35 (0.72)		
Work experience		*F*(3,295) = 1.50,0.21	*F*(3,31) = 1.50,0.23		*F*(3,313) = 1.74,0.16	*F*(3,31) = 0.84,0.48
< 3 months	67.14 (17.29)			3–60 (0.52)		
> 3 months < 1 year	63.00 (28.27)			3.67 (0.49)		
1 to 5 years	52.04 (25.92)			3.45 (0.65)		
> 5 years	56.89 (24.89)			3.34 (0.75)		
Period of employment		*F*(3,295) = 1.34,0.26	*F*(3,31) = 1.34,0.28		*F*(3,313) = 2.99,**0.03**	*F*(3,31) = 1.45,0.25
< 3 months	50.00 (25.17)			3.60 (0.52)		
> 3 months < 1 year	62.35 (23.59)			3.59 (0.66)		
1 to 5 years	53.10 (25.86)			3.48 (0.66)		
> 5 years	56.93 (25.12)			3.29 (0.75)		

Statistically significant results are highlighted in bold

### Patient safety

Patient safety was rated by 318 participants. The mean score was 3.34 (*SD =* 0.72). According to the ANOVAs, nurses perceived a significantly lower level of patient safety than physicians. Moreover, we found that groups significantly differed depending on their periods of employment. Patient safety did not differ as a function of gender, age groups, leadership roles, and work experiences (Table 3).

### Regression analyses

Results of Pearson correlation analyses showed moderate correlations between the three measures to assess inter-professional teamwork (Table 4). There was no indication of multicollinearity. Results of Spearman-Rho correlation analyses for patient safety were similar to those reported in Table 4 (with FAT-K: r = 0.43, *p*<0.001; with NWI-R: r = 0.30, *p*<0.001; with WIP: r = -0.45, *p*<0.001; with SJT: r = -0.01, *p* = 0.86).

**Table 4:** Pearson correlation coefficients (r) for constructs of interest.

	Goal orientation (FAT-K)	Collaboration between nurses and physicians (NWI-R)	Knowledge integration problems (WIP)	Safety-related behavior (SJT)	Patient Safety(HSPSC-D Item)
Goal orientation (FAT-K)	1				
Collaboration between nurses and physicians (NWI-R)	**0.29 (*p*<0.001)**	1			
Knowledge integration problems (WIP)	**-0.34 (*p*<0.001)**	**-0.53 (*p*<0.001)**	1		
Safety-related behavior (SJT)	-0.05 (*p* = 0.44)	0.02 (*p* = 0.76)	-0.04 (*p* = 0.54)	1	
Patient Safety(HSPSC-D Item)	**0.43 (*p*<0.001)**	**0.27 (*p*<0.001)**	**-0.40 (*p*<0.001)**	**-0.01 (*p* = 0.84)**	1

Statistically significant results are highlighted in bold.

Table 5 shows the results of the regression analyses. After controlling for age, gender, and profession, goal orientation and knowledge integration problems were associated with patient safety. Also, nurses reported lower patient safety than physicians. The collaboration between nurses and physicians was not associated with any of the outcomes.

**Table 5:** Linear regression analyses.

Predictors	Safety-related behavior				Patient safety			
	b	SE	*p*≤	Median R^2^ (range)	b	SE	*p*≤	Median R^2^ (range)
				0.03 (0.02 to 0.03)				0.31 (0.30 to 0.32)
Intercept	68.85	15.31	0.001		2.60	0.34	0.001	
Age ^a^								
31–40 years	2.42	3.75	0.52		0.00	0.09	0.98	
41–50 years	0.08	4.30	0.99		-0.18	0.10	0.06	
>50 years	-3.90	4.35	0.37		-0.22	0.10	0.06	
Male gender	5.96	3.76	0.11		-0.08	0.09	0.37	
Profession^b^								
Nurse	-0.30	3.85	0.94		-0.30	0.09	**0.001**	
Other	1.81	6.15	0.77		-0.25	0.14	0.08	
FAT-K	-2.18	2.13	0.31		0.30	0.05	**0.001**	
NWI-R	-0.02	2.56	0.99		0.03	0.06	0.67	
WIP	-2.26	2.75	0.41		-0.26	0.06	**0.001**	

SE = Standard error.

Reference categories

^a^Age <30

^b^Physicians.

Statistically significant results are highlighted in bold.

### Sensitivity analyses

Pearson Correlation analyses on the individual level did only vary slightly from the results reported in Table 4. We provide the results in S1 Table.

As for the regression analyses, results on the individual level showed that collaboration between nurses and physicians influenced patient safety on the individual level, but had no impact on the analyses that took into account hospital ward affiliation. This corresponds with our finding that almost one third of the variability in this outcome was explained by hospital ward affiliation (see Table 3). We provide the results in S2 Table.

## Discussion

This is the first study to systematically investigate inter-professional teamwork, safety-related behavior, and patient safety in German hospitals from the team members’ point of view. Moreover, we examined the association between inter-professional teamwork, and safety-related behavior to take the WHO stated aspect into account. In addition, we investigated the association between inter-professional teamwork, and patient safety.

Results showed that in contrast to former research [32], members of inpatient care teams in our sample perceived inter-professional teamwork in their wards on a level between moderate and high. Our study participants seemed to identify with team goals, had a good collaboration, and few knowledge integration problems. Moreover, in line with the literature [31, 32, 48], physicians in our sample assessed inter-professional teamwork in their wards to be more positive than nurses, which may be explained by different experiences, values, approaches to problem solving, and power between professions [49]. This discrepancy is alarming and can lead to a vicious circle: nurses may not express concerns due to the perceived bad collaboration and physicians may not ask for additional information because they do not perceive the suboptimal collaboration. In addition, members of inpatient care teams in our sample tended to experience their work as less collaborative the longer they worked in their professions, and in the hospital. This may be due to their experience accumulated over time. These differences between the groups were not significant, when we adjusted for clustering. Thus, it appeared that for example the impact of ward affiliation is stronger than professional affiliation. Perceptions of inter-professional teamwork within wards seemed to be similar across professional groups. Due to the impact of ward affiliation, it is therefore advisable to train whole inter-professional teams in future interventions.

Results of the SJT showed that participants reached rather high values in safety-related behavior. About 50% scored between 40 and 75. In addition, the standard deviation was high, which indicated a large variance of data. According to the literature, the recommended response in our SJT is to speak up, share concerns with the team, and initiate clarification for further procedures [42]. About 23% of the participants would act according to this current state of research, which suggests that members of inpatient care teams in our sample had no common approach to deal with doubts about diagnoses. The missing integration of various perspectives (e.g., shared mental models) might entail a risk for patients’ safety.

Members of inpatient care teams in our sample rated patient safety in their wards on a moderate level. This finding should be interpreted with caution since we used a global item not asking for details on patient safety. Similar to our findings in the area of inter-professional teamwork, participants tended to rate patient safety lower the longer they worked in the hospital, which may be explained by their gained work experience. Moreover, physicians in our sample also assessed patient safety to be higher than nurses. Therefore, we suggest nurses and physicians share their differing perspectives and concerns with each other in order to create a shared mental model [13, 14]. As this shared mental model contributes to safer health care for their patients. This should also be a taken up for interventions in the future.

In our study, inter-professional teamwork was not associated with safety-related behavior. One reason may be the low variability in participants’ responses. On a methodological level, our results indicate that the situational judgement test did not differentiate well between different behaviors. Moreover, according to the Theory of Planned Behavior [50], an attitude (here: perceived level of inter-professional teamwork) is not sufficient to form an intention to act (here: intention to speak up). Rather, people’s intention to act depends on other attitudes, subjective norms, and perceived behavioral control. This study did not capture comprehensively those elements. However, if we take the uncovered relationship between the level of perceived inter-professional teamwork and behavior intention at face value, team members of inpatient care teams were convinced of the high quality of inter-professional teamwork, and thus trust in the diagnosis. Consequently, they did not see a need to speak up. This points to the double-edged impact of team trust on patient safety: high trust and satisfaction among team members may impede speaking up, whereas under slightly different circumstances high trust, and satisfaction in the team foster speaking up and thus patient safety.

Lastly, inter-professional teamwork was associated with patient safety in our sample, which is in accordance with current literature [22, 23, 27]. This finding points out the need to continuously improve patient safety by various interventions in the future. Here, too, compared to physicians, nurses perceived a lower level of patient safety, which may be explained by different professions-specific perspectives on patients and treatments. Moreover, the extended time nurses spend with patients may have an impact. The result, that the collaboration between nurses and physicians had an influence on patient safety only on the individual level, may be explained by the different distribution of nurses and physicians working in the hospital wards.

### Limitations

The interpretation of the results is limited by a number of issues. First, selection bias might have occurred in the recruiting process of the KOMPAS project. Since participation was voluntary, we cannot rule out the possibility that inpatient care teams with low levels of inter-professional teamwork were less willing to participate in the study, and we especially reached motivated and well-functioning inpatient care teams, which are interested in continuous improvements of their patient safety skills and behaviors. Second, we used categorical variables to measure work experience. It is not possible to break down the specific number of years participants work in their profession. Due to the result that the >5 years group represented more than two thirds of the study sample, it would be instructive to describe this group in more detail. Further research should use metrical data. Third, the study was cross-sectional, which did not allow statements about causality. Fourth, generalization is limited due to the exclusion of pediatric, emergency, and intensive care wards as well as the moderate response rate. However, low response rates are common problems in clinical setting because of staff shortage, high work load, and absence due to illness. However, we aimed to foster generalizability of the findings by including hospitals from different regions in Germany. Fifth, interpretation of variance analyses is limited because of the unequal group sizes in each category. Sixth, some methodological issues may have occurred due to the way of measuring inter-professional teamwork, safety-related behavior, and patient safety. We used self-reports to assess these variables, which may have caused common method bias. It cannot be ruled out that participants tried to maintain consistency in their responses or tended to respond with a sweeping statement [51]. Moreover, we measured patient safety using one ordinally scaled item, possibly restricting the variability of participants’ responses. Future research should use further more objectively measured outcomes to assess patient safety. Even though SJTs are a common approach in this field, and in addition, we developed our specific SJT based on current literature and experts’ opinions (e.g., physicians, nurses, therapists), and ran a pretest, it is not a psychometrically validated instrument.

## Conclusions

The first important result of our study suggests that higher inter-professional teamwork was associated with a better perceived level of patient safety in German hospitals. On that basis, we recommend the implementation of team interventions to improve patient safety. The second important finding is the impact of professional group, work experience, and period of employment on the perception of inter-professional teamwork, and patient safety. Even though differences between the groups for inter-professional teamwork did not persist after we adjusted for ward affiliation, this result may have implications for practice. Further research should ask the persons involved for their reasons underlying their more critical views within qualitative research in order to profit from their experience. Moreover, it is crucial to encourage these persons to discuss their underlying concerns in the work routine, which could have an impact on patient safety. In this sense, they could serve as role models for less experienced colleagues. This could be an important step on the way from blame culture to safety culture.

To gain a deeper understanding of our findings, we recommend further studies using longitudinally collected data to reveal causal relationships. Moreover, qualitative studies, which capture the rich qualitative aspects of inter-professional teamwork should be conducted. In addition, basic research should investigate the relationship between conditions regarding structural level/ process level, and inter-professional teamwork (e.g., How to organize shift work?, What is the maximum proportion on trainees not compromising teamwork?, Do inter-professional rounds foster inter-professional teamwork?) in order to determine concrete areas for potential improvements, and build a safer health care. Moreover, additional research is required to examine further patient safety predictors.

## Supporting information

S1 Fig Situational judgement test. (TIF)

S1 Table Pearson correlation coefficients (r) for constructs of interest with cluster-mean centered data. (DOCX)

S2 Table Linear regression analyses with cluster-mean centered data. (DOCX)
