# Supplementary figures and images for: Inter-professional teamwork and its association with patient safety in German hospitals—A cross sectional study
Source: PLoS One. 2020 May 29;15(5):e0233766. doi: 10.1371/journal.pone.0233766 (PMC7259596; doi:10.1371/journal.pone.0233766)

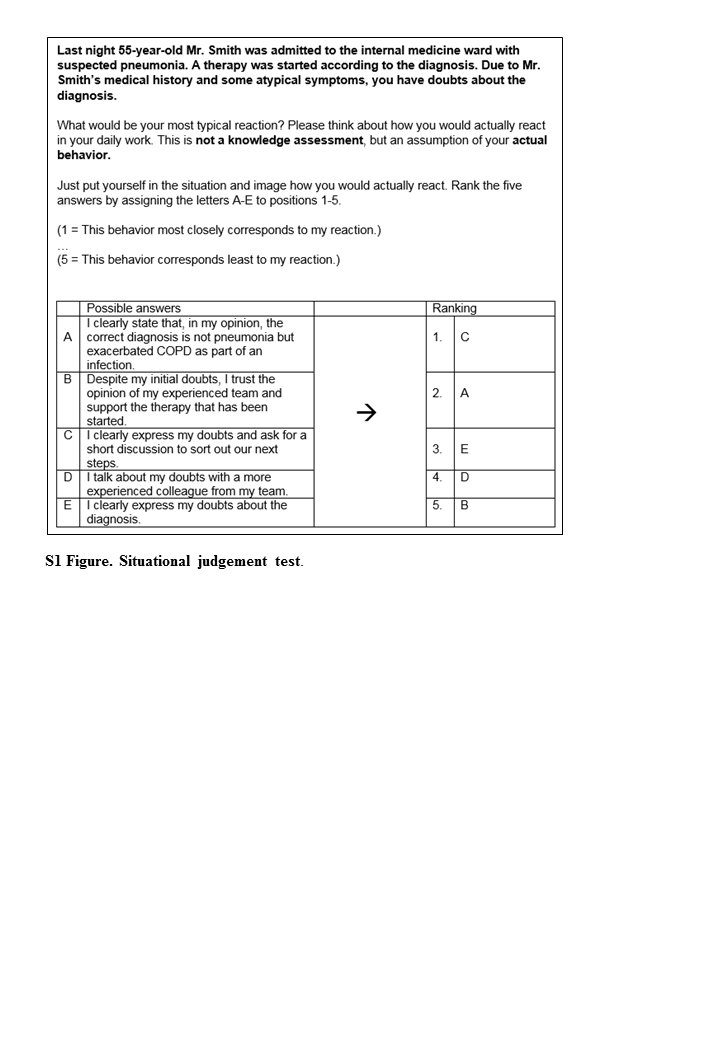

Supplement: S1 Fig — (TIF) [file pone.0233766.s001.tif]
