# Supplementary material for: Inter-professional teamwork and its association with patient safety in German hospitals—A cross sectional study
Source: PLoS One. 2020 May 29;15(5):e0233766. doi: 10.1371/journal.pone.0233766 (PMC7259596; doi:10.1371/journal.pone.0233766)
Supplement: S1 Table — (DOCX) [file pone.0233766.s002.docx]

**S2 Table.** **Pearson correlation coefficients (r) for constructs of interest with cluster-mean centered data.**

|  | Goal orientation (FAT-K) | Collaboration between nurses and physicians  (NWI-R) | Knowledge integration problems (WIP) | Safety-related behavior (SJT) | Patient Safety  (HSPSC-D Item) |
| --- | --- | --- | --- | --- | --- |
| Goal orientation (FAT-K) | 1 |  |  |  |  |
| Collaboration between nurses and physicians  (NWI-R) | **0.31**  **(*p*<0.001)** | 1 |  |  |  |
| Knowledge integration problems (WIP) | **-0.36**  **(*p*<0.001)** | **-0.42**  **(*p*<0.001)** | 1 |  |  |
| Safety-related behavior (SJT) | -0.05  (*p*=0.42) | 0.03  (*p*=0.61) | -0.05  (*p*=0.43) | 1 |  |
| Patient Safety  (HSPSC-D Item) | **0.39**  **(*p*<0.001)** | **0.32**  **(*p*<0.001)** | **-0.41**  **(*p*<0.001)** | -0.02  (*p*=0.78) | 1 |

*Notes*. Statistically significant results are highlighted in bold.
