# Supplementary material for: Inter-professional teamwork and its association with patient safety in German hospitals—A cross sectional study
Source: PLoS One. 2020 May 29;15(5):e0233766. doi: 10.1371/journal.pone.0233766 (PMC7259596; doi:10.1371/journal.pone.0233766)
Supplement: S2 Table — (DOCX) [file pone.0233766.s003.docx]

**S3 Table.** **Linear regression analyses with cluster-mean centered data.**

| **Predictors** | **Safety-related behavior** | | | | **Patient safety** | | | |
| --- | --- | --- | --- | --- | --- | --- | --- | --- |
|  | **b** | **SE** | ***p≤*** | **Median R^2^**  **(range)** | **b** | **SE** | ***p≤*** | **Median R^2^**  **(range)** |
|  | | | | 0.03  (0.02 to 0.05) |  | | | 0.28  (0.28 to 0.29) |
| Intercept | -1.94 | 4.02 | 0.63 |  | 0.24 | 0.09 | 0.006 |  |
| Age^a^ |  |  |  |  |  |  |  |  |
| 31-40 years | 2.52 | 3.77 | 0.50 |  | -0.02 | 0.08 | 0.81 |  |
| 41-50 years | 0.02 | 4.19 | 0.99 |  | -0.13 | 0.09 | 0.14 |  |
| >50 years | -3.76 | 4.35 | 0.39 |  | -0.19 | 0.09 | 0.05 |  |
| Male gender | 6.10 | 3.68 | 0.10 |  | -0.07 | 0.08 | 0.36 |  |
| Profession^b^ |  |  |  |  |  |  |  |  |
| Nurse | 0.30 | 3.76 | 0.94 |  | -0.20 | 0.08 | **0.02** |  |
| Other | 1.94 | 6.20 | 0.75 |  | -0.18 | 0.13 | 0.17 |  |
| FAT-K | -2.97 | 2.46 | 0.23 |  | 0.25 | 0.05 | **0.001** |  |
| NWI-R | 0.75 | 3.19 | 0.81 |  | 0.13 | 0.06 | **0.04** |  |
| WIP | -3.16 | 3.16 | 0.32 |  | -0.28 | 0.06 | **0.001** |  |

*Notes*. SE=Standard error.

Reference categories: ^a^Age <30, ^b^Physicians.

Statistically significant results are highlighted in bold.
